# Variation, mosaicism and degeneracy in the hominin foot

**DOI:** 10.1017/ehs.2021.50

**Published:** 2021-12-27

**Authors:** J. McClymont, K. Davids, R.H. Crompton

**Affiliations:** 1 University of Liverpool, Liverpool, UK; 2 Sheffield Hallam University, Sheffield, UK

**Keywords:** Foot, degeneracy, variability, biomechanics, locomotion, plasticity

## Abstract

The fossil record is scarce and incomplete by nature. Animals and ecological processes devour soft tissue and important bony details over time and, when the dust settles, we are faced with a patchy record full of variation. Fossil taxa are usually defined by craniodental characteristics, so unless postcranial bones are found associated with a skull, assignment to taxon is unstable. Naming a locomotor category based on fossil bone morphology by analogy to living hominoids is not uncommon, and when no single locomotor label fits, postcrania are often described as exhibiting a ‘mosaic’ of traits. Here, we contend that the unavoidable variation that characterises the fossil record can be described far more rigorously based on extensive work in human neurobiology and neuroanatomy, movement sciences and motor control and biomechanics research. In neurobiology, *degeneracy* is a natural mechanism of adaptation allowing system elements that are structurally different to perform the same function. This concept differs from *redundancy* as understood in engineering, where the same function is performed by identical elements. Assuming degeneracy, structurally different elements are able to produce different outputs in a range of environmental contexts, favouring ecological robusticity by enabling adaptations. Furthermore, as degeneracy extends to genome level, genetic variation is sustained, so that genes which might benefit an organism in a different environment remain part of the genome, favouring species’ evolvability.

## Graphical abstract

**Figure:**
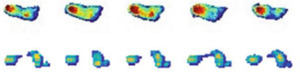

**Social media summary:** Limb-bone variation is explicable by the recombination of a set of bones to produce different outputs in each activity.

## 1. Introduction

Walking – as with all biological actions – is characterised by functional variability (Arutyunyan et al., 1968; Davids et al., 2003). In the living great apes and by extension in our fossil ancestors, a variable array of locomotor behaviour is evident, from bipedal walking on the ground and in trees, to swinging, leaping, vertical climbing, knucklewalking and more. While our habitual locomotor mode is bipedal, we have the ability to perform all of the above-mentioned behaviours: while most of us would fail in an attempt to brachiate on a ‘jungle gym’, consider a parkour athlete or gymnast, or a human baby's ontogenetic climbing phase. Equally, many of us might have seen the widely available video footage of *Gorilla gorilla* casually standing or walking upright. Not only is there high inter-species variability in locomotor mode, but inter- and-intra-individual variability in extant and extinct great apes is also staggeringly high. Despite the omnipresence of this natural, functional feature of movement, palaeoanthropology lacks a strong theoretical framework anchored in biomechanics, or complex-adaptive-systems biology, from which to interpret variability. As a consequence, a long-running debate regarding the significance of the taxonomic and morphological variability in the fossil record has ensued (Wood and Boyle, 2016). Calls to further understand variation in locomotion in extant great apes can no longer be ignored (Crompton et al., 2003; Alexander, 2013; D'Août & Aerts, 2008; Carlson et al., 2011; Neufuss et al., 2014; McClymont et al., 2016; McClymont & Crompton, 2021).

The phenotypic morphology of bones reflects habitual movement patterns practised during life (Bock, 1965), and this relationship between movement and bone shape is a fundamental interpretive paradigm in bioarchaeology and palaeoanthropology (Jungers and Minns, 1979; Trinkaus & Ruff, 1999; MacLatchy et al., 2000; Madar et al., 2002; Ruff, 2002; Ruff et al., 2006; Boyle et al., 2018). Fossil hand and foot bones are rare, but they can be useful for interpreting the evolution of locomotion as they are the primary biomechanical unit to interact with the environment (reviewed e.g. in Klenerman and Wood, 2006; D'Août and Aerts, 2008; Stolwijk et al., 2013; Vereecke et al., 2008). Analysis of bone shape in isolation, however, does not definitively test behaviour or locomotor mode, as relative contributions of soft tissues, relationships between applied forces and the dynamics of the system cannot be accounted for. Given the truly fragmentary nature of the fossil record, it is not uncommon to find that only one skeletal element is available to characterise taxa and locomotor behaviours. More often than not the variability and overlap in bone shape between taxa are noted but is not elaborated on.

The current tradition is that fossil foot elements represent a ‘mosaic’ picture of foot evolution from 7 million years ago (Ma) to the present, enabling locomotor behaviour in both terrestrial and arboreal environments (Zipfel et al., 2011; Haile-Selassie et al., 2012; DeSilva et al., 2013). Complex palaeoenvironments would exert selective pressure for locomotor plasticity to exploit varied, unstable and rapidly changing microhabitats, with hominids displaying a combination of plesiomorphic and apomorphic characteristics (Senut et al., 2001; Lovejoy et al., 2009a, b). As the fossil record has expanded, author interpretations have uncovered functional overlap in the foot extending from the early Pliocene (circa 4.4 Ma) to the late Miocene (circa 7 Ma), reflecting this apparent plasticity and diversity in locomotor behaviour.

Plasticity hypotheses are long established in evolutionary biology (Via & Lande 1985; Schlichting & Pigliucchi 1993; Stearns, 1989; Odling-Smee & Braithwaite, 2003; Laland & Sterelny, 2006; Pigliucci, 2009; Pigliucci & Müller, 2010; Danchin et al., 2011; Laland et al., 2015) but not in hominin palaeontology, owing probably to the contention over the role that plasticity plays through phenotypic and genetic accommodation (Pfennig and McGee, 2010; Moczek et al., 2011), i.e. in palaeoanthropology the adaptation of parts in response to environmental constraints is independent of genetics. However, plasticity advances diversity in niche construction in unique environments (Odling-Smee & Braithwaite, 2003) and encourages population-level connectivity and gene flow (Crispo & Chapman, 2008). It increases the probability of shifts in adaptive peaks, radiations and speciation events (Price, 2005; Lande, 2009), contributing to the obvious spatio-temporal variation in selection parameters (Huey et al., 2003; Duckworth, 2009; Cornwallis & Uller, 2010). In a review on environmental constraints that shape human life-history variation, Kuzawa and Bragg (2012) observed that many of the life history traits they examined (including low birth rate, delayed maturity and high fertility), exhibited high phenotypic variation stemming from developmental and behavioural plasticity. These traits were in response to environmental factors such as accidental death or nutrition stress. While the idea of locomotor plasticity has been touched on in the palaeontology literature (Venkataraman et al., 2013a, b; Boyle et al., 2018; Crompton et al., 2021; McClymont & Crompton, 2021), we believe that future studies regarding variation could be firmly conceptualised on the theoretical paradigm of degeneracy, which we elucidate in this position paper.

## 2. Functional redundancy and degeneracy

In 1962, an English translation of a 1933 Russian volume by influential movement scientist Nikolai Bernstein was published. He began with the observation that many more kinematic degrees of freedom existed in the locomotor system than were required to perform its habitual activities: in other words, the same motion could be achieved with much less complexity of joint arrangement. This was referred to as ‘redundancy’ after engineering design, where potential failure of individual elements is often offset by inclusion of duplicate elements which are ‘redundant’, unless failure occurs elsewhere. The hands and feet provide an excellent example of this, together containing more than 50 bones. The joints between them are complex and multifaceted, crossed by dozens of ligaments and tendons. They are, therefore, typified by an excess of degrees of freedom, or potential ways that joints can interact to produce the appropriate motion for each step. However, unlike redundancy in engineering, in biological systems, elements are structurally different, and can perform the same function. They can also form different combinations, underlying different functions (Tononi et al., 1999). Such systems comprising multifunctional elements are thus described as ‘degenerate’: systems may thus be characterised by low or high degeneracy.

### What is degeneracy?

Unlike redundant systems, degenerate components can produce different outputs while satisfying different performance and environmental constraints (Edelman & Gally, 2001). A degenerate system can vary the way that different components produce the same functional output and is highly adaptive in response to unpredictable changes in the dynamic environmental conditions underpinning movement. Degeneracy is an inseparable characteristic of biological systems and is a direct consequence of natural selection (Whitaker & Bender, 2009). Degeneracy operates from the molecular (McClellan, 2000) to the gene network level (Dudkiewicz et al., 2005) and is a primary property of neural networks (Tononi et al., 1999) and immune system response (Edelman, 1974).

It is fundamental to understanding movement coordination and its acquisition (Chow et al., 2009; Davids and Glazier, 2010; Seifert et al., 2016), promoting flexibility and stability during complex motor learning (Komar et al., 2015), and is thus a key property of functional adaptations to tasks and environments. In the domain of movement, degeneracy supports a neurobiological system to organise highly varied movement behaviours, without compromising the original function of the skeletal structure (Edelman & Gally, 2001; Price & Friston, 2002; Mason, 2010).

We can illustrate degeneracy in modern humans with reference to the ideas of DeSilva (2009). In this paper, data from chimpanzee and human distal tibial morphology was presented to argue that human morphology could not sustain sufficient dorsiflexion to allow chimpanzee-like vertical climbing. A later and largely ecomorphology focussed study found that indigenous human arboreal foragers do produce chimpanzee-like kinematics in vertical climbing (Venkataraman et al., 2013a, b). Subsequently, Venkataraman and DeSilva combined their individual approaches with field ultrasound techniques, establishing that changes in fibre length of gastrocnemius in habitual arboreal foragers adequately offset any derived features of the ankle associated with bipedalism (Venkataraman et al., 2013b). We argue that the range of gastrocnemius fibre length provides evidence of degeneracy in ourselves. Myatt et al. (2011, 2012) reported another example, demonstrating that the ranges of muscle cross-sectional area and fibre lengths of great apes, in general, overlap considerably with each other, implying that their length/tension and torque/joint angle capacities also overlap, consistent with degenerate function in hominoids as a whole. Amongst the many sources of variation, Boyle et al. (2018) noted the well-established contribution of muscles lost in most individuals of living species to intra-species variation. It remains to be shown, however, what genetic and/or developmental mechanisms drive this and other sources of variation.

Next, we contextualise mosaicism in reference to fossil feet and footprints, with specific reference to the way variance and invariance are interpreted in evolutionary biomechanics. This outline is followed by a discussion of the role functional variability plays in locomotion, and finally we discuss the relationship of degeneracy to evolution and adaptation. We do not wish to devalue any previous locomotor assumptions that have accompanied interpretations of fossil bone morphology, but rather to suggest that the degeneracy framework would provide more robust interpretation of observed variance, and of any locomotor assumptions, supported by palaeoenvironmental data and the ecomorphology framework (Wainwright, 1991).

## 3. Mosaicism

Mosaicism is a common term in palaeoanthropology, first introduced in reference to *Archaeopteryx* (de Beer, 1954). It is applied to all anatomical regions (e.g. in the recent discussion of the *Homo longi* skull from Harbin (Ji et al., 2021) which is described as having: ‘a mosaic combination of plesiomorphic and apomorphic features’ (Ji et al., 2021, p. 1)). One of the earliest mentions of mosaicism in human evolution was by Day and Napier (1964), referring to the ‘*Homo habilis*’ OH8 foot bones and OH7 hand bones from Olduvai Bed 1. Oxnard and Lisowski (1980) disputed the conclusion of Day and Wood (1968) that the OH8 ‘*Homo habilis*’ foot possessed human-like arches, and belonged to a biped, if not a striding biped, like us. They argued that the articular surfaces of the bones suggested that an arch was absent. Later, Kidd et al. (1996) used the term mosaic, specifically to refer to function of the OH8 foot evidenced by multivariate analyses of ‘biomechanically relevant features’ (Kidd et al., 1996, p. 269). Discussions surrounding the *Australopithecus sediba*, and the much later *Homo naledi* feet, make extensive use of the term mosaicism, suggesting the individual may have practised a ‘unique form of bipedalism with some degree of arboreality’ (Zipfel et al 2011, p. 1417). For the foot of *Homo naledi*, Harcourt-Smith et al. (2015, p. 1) state that it is ‘a unique mosaic previously unknown in the human fossil record’.

This tradition seems to combine uses of mosaicism to describe a combination of different functional features or adaptations. For the *A. sediba* hand, Kivell et al. (2011) continue the tradition of functional mosaicism between features which could be seen as adaptive for arboreal locomotion (such as curved digits) and features which might be seen as adaptive for a precision grip and perhaps toolmaking, such as shorter fingers and longer thumb. They make brief mention of the much earlier StW573, which also combines these manual features. Kivell (2015), while not using the term mosaicism, argues that the *H. naledi* hand shows yet another distinct combination of morphological features not yet known in any other hominin.

In the above, joint shape and bone proportions are regarded as crucial evidence for function in both the hands and feet. Particularly in more recent papers, these anatomical features are held to indicate differences in bipedalism and most often differences in arboreal vs. terrestrial adaptation. There is an interplay between perceived locomotor similarity to humans in some features, but dissimilarity in others, and the concept that mosaicism exists in the degree of ‘hominisation’, and the extent to which features are plesiomorphic or apomorphic. In a helpful review of mosaicism, Parravicini and Pievani (2019) acknowledged this interplay by recognising that their ‘Mosaic type 1’ (‘hominin morphological instability’), characterises the complex morphology of the *H. naledi* hand and foot, and interacts with their ‘Mosaic type 2’ (‘multiple phylogenetic trajectories for the same trait’), where *H. naledi*, *A. sediba* and *Australopithecus africanus* are seen as having different kinds of bipedalism. Mosaicism, however, has a far wider application: ‘The concept of “mosaic evolution” […] refuted the notion of harmonious development by affirming that individual organs could have independent phyletic histories, despite the evident correlation of parts within any organism’ (Gould, 1977, p. 234). Parravicini and Pievani (2018) give the example of human language, which is not a single trait but a convergence of different traits with different evolutionary history: this type of mosaicism they term ‘Mosaic Type 3’. Nevertheless, in hominin studies, the term mosaicism continues to be applied particularly to the hand and foot.

A recent review of foot evolution in relation to bipedalism, however, suggests that doubt is beginning to be expressed about the usefulness of the mosaicism concept: DeSilva et al. (2018) conducted an extensive cladistic analysis of foot-bone morphology based on individual bones, reaching similar conclusions to earlier studies on the existence of mosaicism in ‘walking kinematics’ and arboreality in different lineages. We do, however, recognise that particularly given small sample sizes, their cladistic analysis of individual bones may ignore overall equivalence in kinematics, via ‘compensation’ between morphologies of different bony elements. We argue that this is probably the case, and that mosaicism in all three forms simply reflects basic mechanisms of evolvability and ecological robusticity, based on biological variation from the level of the genome all the way to the level of functional behaviour. We propose that mosaicism in the hand and foot is simply a reflection or merely a literal description of the number of bones, the joints they make up and the ligaments and tendons crossing joints, which allow morphologically different bones to contribute to the same external function, and equally to contribute, in different ways, to other external functions.

## 4. How variance and invariance are treated in evolutionary biomechanical interpretations.

Griffin and Tversky (1992) suggested that palaeontological research focusses on the ‘strengths of the extremeness’ of morphological difference, without a strong interpretive paradigm within which to interpret the observed extremeness. Given the depauperate nature of the fossil record, both in representations of species and in representation of the bony elements of individuals, a search for uniqueness has often prevailed over biological balance. This is exemplified by Spoor et al. (2010), in the naming of a new hominin genus and species *Kenyanthropus platyops* based on a single, taphonomically deformed australopith cranium. White (2003) strongly (and we agree correctly) criticised this as an example of a lack of recognition of variation in palaeoanthropology. Surprisingly, however, the genus *Kenyanthropus* continues to be widely recognised. Equally, Brunet et al. (2002) declared the 7 Ma *Sahelanthropus tchadensis* unequivocally hominin, based substantially on foramen magnum angle. This is despite the age of the specimen falling within the most generally accepted age range of the panin–hominin separation, based on molecular evidence (Jensen-Seaman & Hooper-Boyd, 2008). More recently, however, Ruth et al. (2016) reported that foramen magnum angle is not correlated with locomotor behaviour in rodents, strepsirrhine primates or marsupials, but again this has not led to Brunet et al.'s (2002) claim being challenged.

### How philosophy, science and the ‘covering law’ model (mis-)shaped traditional understanding of variability

Assumed invariance is the product of a tendency towards biological determinism (Lewontin, 2001) that has traditionally constrained the interpretation of behavioural interpretations in palaeoanthropology. It is ironic that Darwin paid a significant amount of attention in his writings on evolutionary theory to reducing the emphasis on a ‘group modal’ perspective, in favour of a focus on variability *within* species (Darwin, 1871). The lack of recognition of variability in palaeoanthropology recalls the Platonic ‘ideal’, whereby variation was long regarded as pathology. This conceptualisation of variability has shaped theoretical and scientific understanding, adhering to the philosophy of ancient Greece, informed by Plato, Aristotle and Socrates. To exemplify, Plato's dialogue, The Meno, seeks to explain knowledge, experience and understanding in humans with reference to internalisation of *universals*, *kinds*, *types* and *templates* which are used to organise meaning in the world. This philosophical approach has led to the dominance of the ‘*covering-law*’ model in theory and science, which emphasises the labelling and generic categorisation of phenomena in groups, overlooking the relevance and pertinence of contextually driven variability. Criticisms of the way that the covering law model neglected and mis-represented variability in complex systems are relevant and influential because they tend to ‘dump context in favour of universals’ (Riley & Turvey, 2004, p. 164). Riley and Turvey (2004) correctly criticised this, because it neglects emergence in nonlinear phenomena (such as evolving limbs and bone structures) whose trajectories are historically dependent on initial conditions, context and discontinuities arising from constraints on their flow (as information constraining system dynamics of long timescales).

To exemplify the dominant influence of the covering law model, in 1935, a ‘classic’ study by Elftman and Manter claimed that there was a clear distinction between the feet of humans and those of other apes (represented only by a chimpanzee), with only the latter declared to have clear ground contact in the lateral midfoot (now termed a ‘mid-tarsal break’), and so, a more posterior push-off than humans. This led to the Root model (Root et al., 1977) of an ideal ‘*pes normalis*’ with a strongly expressed and largely permanent arch, stiff lateral midfoot and predominant hallucal toe-off. This model was later elaborated by Bojsen-Møller (1979), who identified an interlocking mechanism of the cuboid peg matching a groove on the plantar calcaneus in humans, supporting a bony lateral arch. Neither hypothesis was tested *in vivo*, but both became embedded in podiatric, orthopaedic, anatomical and palaeoanthropological training, whereby performance departures from that ‘ideal’ were long assumed to be dysfunctional.

### Disambiguating the Root model

Over the last 10 years the weight of evidence from experimental biomechanics has convinced many academics working with feet that the Root model is largely incorrect, and the vast variability in biomechanical parameters when testing the foot is relevant and significant to understanding of how functionality is driven by contextual constraints. This omission is now being addressed slowly but surely (see e.g. Nester et al., 2007a, b; Lundgren et al., 2008, McClymont et al., 2016; Morrison et al., 2017, 2018; Price et al., 2018). *In vivo* invasive kinematics and cadaver studies of foot bone motion have captured high variability in the range of motion in midfoot joints. Lundgren et al. (2008) found so much functional variability in a sample of just six individuals that some had higher mobility proximal to the cuboid, while in others, higher mobility was distal to it. This level of inter-individual variability in movement coordination has increasingly been observed and conceptualised in the movement sciences and biomechanics (see Glazier & Davids, 2009a, b). Midfoot flexibility in humans has been discussed in the orthopaedic literature, noting high flexion in the sagittal plane (Ouzounian & Shereff, 1989; Whittaker et al., 2011), while substantial midfoot dorsi-flexion, indicating internal variation in midfoot mobility, is widely accepted in biomechanics (see e.g. Lundberg et al., 1989a–c; Stacoff et al., 2000; Arndt et al., 2004; Arndt et al., 2007; Lundgren et al., 2008). Caravaggi et al. (2016) extended this concept further, towards foot–ground interaction, by demonstrating a statistical link between internal foot bone motion and plantar pressure (that is the pressure exerted externally against the substrate) and identified a high, but unexplained variance in plantar pressure.

While the Root model is now largely discredited in podiatric medicine, it has persisted within palaeoanthropology along with the treatment of variation as an unexplainable obstacle. For example, citing the Elftman–Manter/Bojsen–Møller/Root model, Jungers et al. (2009) claim that limited saliency in the cuboid peg from the LB2 *Homo floriesiensis* skeleton implies bipedal gait distinct from our own. Meldrum et al. (2011) claim that a line in a single footprint in the Laetoli G-1 trail (G-1/26) is evidence of a mid-tarsal break in *Australopithecus afarensis.* Contrarily, Ward et al. (2011) interpreted features of a single fossil fourth metatarsal element of *A. afarensis* and the orientation of the proximal and distal ends, easily overlapping the human range, but not that of *Pan* or *Gorilla*, as showing proximo-distal torsion along the diaphysis. Furthermore, they interpreted its deep flat base and cuboid facets as demonstrating that the foot was functionally unable to produce a ‘midtarsal break’. Again, DeSilva et al. (2010) studied the cuboid facets of StW 485 and StW 596 (two isolated MT4 elements from Sterkfontein), and described them as flat and distinctly different from the convex facet of African apes. From this comparison, they claim that this feature reflects a lack of midfoot compliance (as seen in chimpanzees).

Before the recent increase in fossil discoveries, McHenry and Jones (2006) quantified the variability of great toe adduction via analysis of the encroachment of the metatarsal 1 facet on the medial cuneiform, both in extant hominoids and in two fossil hominins. Despite a hominin sample size of 2, the authors concluded that hominins were specialised for bipedalism based on their highly adducted and unopposable great toe. However, Zipfel et al. (2011) compared the calcaneal morphology of the foot and ankle of *A. sediba* with five other great apes (their figure 4B), which latter formed loosely overlapping clusters, and found *A. sediba* to be grouped with the gorillas. The same is the case for a more recent study of the subtalar joint of *A. sediba* compared with eastern and western *Gorilla*, *Pan*, *Hylobates*, *Pongo* and modern *Homo* (Prang, 2016), where a canonical variates analysis of nine talar and calcaneal variables shows a clear overlap in the standard deviations between species. An excellent study by Dunn et al. (2014) documents high variation in western (*Gorilla beringei*) and eastern (*Gorilla gorilla)* talar morphology, suggesting a link between their morphology and their environment. With this notable exception, awareness of variation has otherwise not often been accompanied by a serious attempt to explain and interpret its role in movement, most likely because of a lack of a grounding theory upon which to hang such interpretations.

Caravaggi et al. (2016) demonstrated a direct statistical link between foot pressure topology and foot bone mobility. Mobile foot joints enable the exchange of force between the foot and ground in a smooth ‘roll-over’ motion (Caravaggi et al., 2016). This roll-over has been observed in all great apes studied to date (Bates et al., 2013a). Inter-species comparison of pressure patterns of human, bonobo and orang-utan subjects revealed a clear overlap in the distribution and magnitude of pressure between these hominoid taxa (Vereecke et al., 2003; Crompton et al., 2010; Bates et al., 2013a). Humans, bonobos and orang-utans can all display either a so-called ‘mid-tarsal break’ or very low lateral midfoot pressure, owing to the high range of motion and variability between the tarsals, and between tarsal and metatarsal elements in great ape feet. While the habitual mean tendency of modern human midfoot mechanics is certainly one of a rigid lateral midfoot, compared with a more compliant midfoot in the other African apes, recent datasets have revealed consistently high variability in arch compliance (exhibited by high peak pressure) in the lateral midfoot of humans (Bates et al., 2013a; DeSilva et al., 2013). Bates et al. (2013a) report high lateral midfoot pressures in their human sample, reporting observed overlap in relative midfoot pressure with other species of great ape (*Pan paniscus* and *Pongo pygmaeus*), as previously reported by Vereecke et al. (2003) and Crompton et al. (2010).

Fossil footprint trails have offered some hope of reconstructing the external function of the early hominid locomotor system. A recent contribution by Hatala et al. (2016) based on five of the intact 11 Laetoli G-1 prints (attributed to *A. afarensis*) claimed to identify functionally meaningful differences between the gait of the Laetoli G-1 trackmaker and that of modern humans. Their interpretation is focussed on supposed similarities to footprints made by chimpanzees during bipedal locomotion and they argue that the Laetoli G-1 track maker walked with a more flexed knee posture than modern humans. Crucially, however, as pointed out by Bennett et al. (2016), they did not register the Laetoli, chimpanzee and human footprints to each other, which involves the stacking and alignment of each of the footprints’ topological surfaces, enabling direct, statistically robust, topological comparisons (Pataky & Goulermas, 2008; Pataky et al., 2008; Crompton et al., 2012; Bates et al., 2013b; McClymont et al., 2016). In line with previous studies, Hatala et al. (2016) subjectively selected 14 ‘functional’ points on each plantar surface based on anatomical markers for comparison. Such ‘region of interest’ comparisons are common in the literature (see, e.g. Hughes et al., 1991; Rosenbaum et al., 1994; Zhu et al., 1995; Kernozek et al., 1996; Brown & Mueller, 1998; Drerup et al., 2001; Burnfield et al., 2004; Segal et al., 2004; Taylor et al., 2004; Warren et al., 2004; Yang et al., 2005; Shu et al., 2010; Paton et al., 2011; Barn et al., 2015; Howcroft et al., 2016; Wallace et al., 2021), and while Hatala et al. (2016) chose to compare 14 *points* rather than the three to 10 *units* compared in the above-cited studies, we suggest that the two methods can lead to similar inaccuracies in results. Anatomical masking breaks the plantar surface into subjectively defined regions, reducing the naturally occurring functional appearance of pressure patterns, and fails to account for the plantar surface as it exists in reality, i.e. a single functional unit. Research has shown that masking can conflate or even reverse statistical interpretations when compared with pixel-by-pixel analyses (Pataky et al., 2008). Furthermore, it has been shown that longer walking sequences provide more stable measures of variability (Gök et al., 2002; Barker, 2006) and that a high number of data points or steps is needed in order to capture the naturally occurring levels of habitual variation. McClymont and Crompton (2021) recently demonstrated that some 200 consecutive pressure records, collected from a pressure-sensitive treadmill, are necessary to statistically characterise an individual's habitual plantar pressure pattern. We accept that this requirement is not achievable in many cases given differing access to equipment, age and pathology.

In a recent comparative analysis, we reported a comparative pedobarographic statistical parametric mapping analysis of the footprint trails from the following sites: Laetoli (probably made by *A. afarensis*), Ileret (probably made by early *Homo*), Walvis Bay, Namibia (made by subrecent *Homo sapiens*) and a modern human sample (McClymont et al., 2021). Both the Laetoli and Ileret footprint trails show a significant difference from the modern human sample, evidenced by a deeper impression under the region of the medial arch. Yet when the Laetoli and Ileret prints are compared with the anatomically modern human footprints from Namibia, the Ileret prints show a small area of statistically significant difference in the medial mid-foot, i.e. a deeper impression. However, there are no statistically significant differences when compared with the mid-foot of footprints made by habitually unshod (Namibian) recent humans and our 11-print Laetoli dataset. That differences in relative midfoot footprint depth are greatly reduced (and in the case of Laetoli, eliminated) when early *Homo* (Ileret) and *Australopithecus* (Laetoli) are compared can be understood in the context of a previous study of foot pressures in habitually unshod modern populations. This observation revealed such individuals to have functionally lower medial longitudinal arches on average than modern, Western individuals (Willems et al., 2017). In other words, it appears that habitual shoe-wearing artificially induces a higher medial arch, more similar to the ideal ‘*pes normalis*’ of the Root model.

Bates et al. (2013b) reported that experimental and modelling studies of the relationship of footprint depth to footprint morphology show a clear tendency for deeper prints to have relatively deeper impressions under the forefoot. It is, therefore, likely that the statistically significant differences between the Laetoli G-1 and Ileret prints are attributable to the greater overall footprint depth at Ileret, where moisture content is likely to have been higher and the sediment correspondingly weaker in strength (Bennett et al., 2009). Similarly, the relatively greater number of deep prints from Namibia (i.e. those from wetter substrate contexts) could readily account for a deeper hallux impression than Laetoli. Based on the nature of footprint formation, and the relative depths from each site, we argue that there is no evidence of detectable functional differences in foot (and hence upper body) biomechanics between the Laetoli G-1, Ileret and Namibian footprint trails.

The pixel-level statistical analysis presented here agrees with the finding of Bennett et al. (2016) based on the depth profiling of registered prints. Here, we interpret the lack of any statistically significant differences between fossil footprint sites as evidence of high redundancy and locomotor plasticity in the hominins, driven by neurobiological degeneracy and substrate effects (Bates et al., 2013b; McClymont et al., 2021). The classic interpretation of fossil footprints claims that differences (or invariance) in morphologies between species creates different internal loading patterns, producing a different print topology. Given the overlap between great apes we have noted above in foot pressure and that substrate texture is the prime formative influence in footprint creation, we argue that the functional differences gleaned and extrapolated to lower and upper body posture and locomotor practice (Hatala et al., 2016) are a false positive, i.e. inferring an experimental effect when none actually exists (Pataky et al., 2016), in this case, the error of inferring internal and external mechanical differences from external topology alone.

Given that some 200 walking cycles on a pressure-sensitive treadmill are necessary to characterise an individual's habitual foot pressure (McClymont & Crompton, 2021) and the substrate effects of making footprints noted by Bates et al. (2013b) and Bennett et al. (2016), we regret that there seems little hope of correctly interpreting fine functional details such as midfoot characteristics, or the extent of lateral-to-medial force transfer from early hominin footprints. Only the most unambiguous features of trails are likely to yield high-quality biomechanical signals, e.g. the relatively greater depth of impressions under the heel than the metatarsal heads (forefoot) in Laetoli G-1 does indeed provide a signal of upright locomotion rather than ‘bent-hip–bent-knee’ gait (Crompton et al., 2012). Otherwise, the Laetoli prints can only be taken as reflecting the locomotor plasticity available to the track maker at 3.3 Ma.

A recent review article by Wood and Boyle (2016) suggests clear evidence for species-level distinctions in morphology between, e.g., *Australopithecus anamensis* and *A. afarensis*, but simply calls for palaeontologists not to be ‘over-confident’ in interpretations of fossil remains. Similarly, Ward and Middleton (2016) report more substantial variation in thoracic shape in anthropoids (including humans) than previously expected. They found that the upper rib cage shows only a weak link to locomotion, and the lower rib cage shows a closer link to pelvic morphology than to upper rib cage morphology, and simply conclude that variability needs to be taken into consideration more seriously in studies of locomotor evolution. Again, Marigó et al. (2016) quantified inter-subject variability in the morphology of the calcanei and astragali of a middle Eocene primate, *Anchomomys frontanyensis*, to assess its phylogenetic position compared with other crown strepsirrhines. They demonstrate a large statistical overlap in linear metrics of morphology within crown strepsirrhines, both living and fossil; however, this was not explained further. Similarly, Boyle et al. (2018) showed extreme variability in the lateral plantar process of the calcaneus, overlapping with *G. gorilla*, *P. troglodytes*, *A. sediba and A. afarensi*s (A.L.333-8), concluding that the variability might signal normal variation, locomotor diversity or two independently evolving adaptations for heel strike in two different hominin lineages. More recently, Marigó's team report high variability in humeri of Adapiformes (Eocene primates), concluding that high morphological variability suggests that 14 different locomotor repertoires were used by different species (Marigó et al., 2020). Similarly, Drapeau and Harmon (2013) plot, but do not explain, the considerable overlap of metatarsal head torsion in their dataset of monkeys, non-human apes, humans and australopiths, rather simply attributing variation to differences in locomotion. Tsegai et al. (2018a, b) compared trabecular patterns in humans and chimpanzees, and found that while trabecular bone volume fraction shows differences between forelimb and hindlimb in humans and chimpanzees, this parameter did not clearly reflect locomotor loading, whilst degree of anisotropy was more likely to reflect locomotor loading than species. Care is thus required in interpreting what otherwise appears to be a promising measure by which to relate locomotor activity to species in isolated fossil bones. While in the above studies, variation has at least been acknowledged, the observed variation can be explained by plasticity and degeneracy.

## 5. Locomotor variation

As observed by Bock (1994), robust interpretations of evolutionary adaptations should be ‘nomological’, that is, regardless of system structure or composition, underlying phenomena should be explained by similar abstract principles. For example, the natural laws of physics underlie a system's capacity to maintain stability during dynamic movement. During locomotory activity, natural selection drives the adaptation of interactive hard and soft tissue configurations that enhance performance relative to the constraints of a specific environment, and each may be studied and distinguished between species (Laland et al., 2015). This is one of the core assumptions of the Extended Evolutionary Synthesis, termed ‘reciprocal causation’, whereby: ‘organisms shape, and are shaped’ equally (p. 2, Laland et al., 2015) by selective and developmental environments. Thus, developmental and niche diversification works *with* natural selection in determining the rate and direction of adaptations (Laland et al., 2015). In particular, the relatively new field of ecomorphology seeks to explain the covariation of ecology and morphology (Winkler, 1988; Wainwright, 1991). This field has particularly clear applications to Primates, since they are typically large-bodied and predominantly arboreal, thus, ensuring stability and fall avoidance during locomotion is likely to be naturally selected for in arboreal apes. Vertebrate gait is characterised by variable fluctuations in stride index parameters that functionally stimulate stability during locomotion (Grillner, 1985; Arutyunyan et al., 1968; Davids et al., 1999; Dingwell et al., 2001; Jordan et al., 2007a, b). The rhythmic, alternating activity of the inverted pendulum, for example, becomes a major factor in stability of both bipeds and quadrupeds. Local dynamic stability measured by variability in human walking parameters has been revealed as a predictor of the likelihood of fall injury (Calandre & Conde, 2005; Lockhart & Liu, 2008). It may, thus, reasonably be assumed to be naturally selected for in humans as well. A stable gait is defined as one during which the individual does not fall over, despite perturbations (Dingwell et al., 2001; Bruijn et al., 2013). In primates and other arboreal species, stability of branch support itself is an important parameter to contend with.

Bernstein (1967, p. 234) referred to the importance of movement organisation describing it as ‘repetition without repetition’, in order to enhance the functionality of behaviour. A seminal insight of Bernstein (1967) was that movement organisation is ‘function specific’, not ‘muscle specific’, the latter being a dominant idea in the neuroanatomically dominated Russian movement science theory for many years. According to his insights, movement (re)organisation is temporarily assembled from system degrees of freedom to adaptively (re)produce a specific, intended task outcome, under varying task constraints (Bernstein, 1967; Latash et al., 2002). In all animal locomotion, the coefficients of variation of step-length and step-width change with time, and relative to external and internal constraints (Bernstein, 1967; Alexander, 1984; Bock, 1994), naturally facilitating adaptative stepping. This, step-to-step strategy varies to allow for anticipated or unexpected perturbations and changes in speed, slope, surface and substrate compliance. Thus, each step is uniquely organised to resist internal (Su & Dingwell 2007; Byl & Tedrake 2009) and external perturbations (Harris & Wolpert 1998; Faisal et al., 2008). The standard coefficient of variation equation (CV *=α/μ*), however, is not a measure of functional stability in a moving system (Dingwell et al., 2001; Bruijn et al., 2013; van Emmerik et al., 2016), rather, the distribution (or range) of a parameter about the mean. Differences in the coefficient of variation of step kinematics are relatively small: for example, only a few per cent increase or decrease in parameter change will occur, signifying the finely tuned nature of motor control mechanisms that regulate the basis of an uninterrupted, continuous gait pattern (Gabell & Nayak, 1984; Hausdorff et al., 1997; Alexander, 2013; Terrier and Schutz, 2005; Jordan et al., 2007a, b; Vaillancourt et al., 2004).

We have noted that in palaeoanthropology a change in midfoot function is often regarded as the hallmark of human evolution: the transition to terrestrial bipedality (e.g. Ward et al., 2011). As humans walk faster, approaching the walk–run transition at some 1.88 m s^−1^, the plantar aponeurosis (apparently a derived feature in hominins, see e.g. Crompton et al., 2010), pre-tenses prior to heel strike, collapsing through mid-stance and reaching maximum tension through to late stance phase (Caravaggi et al., 2016). The arch-stiffening mechanism is speed dependent, but this feature does not appear to be reflected by variability in plantar pressure, which has been shown to be independent of speed (McClymont et al., 2016).

## 6. Degeneracy and evolution

**Figure 1.**
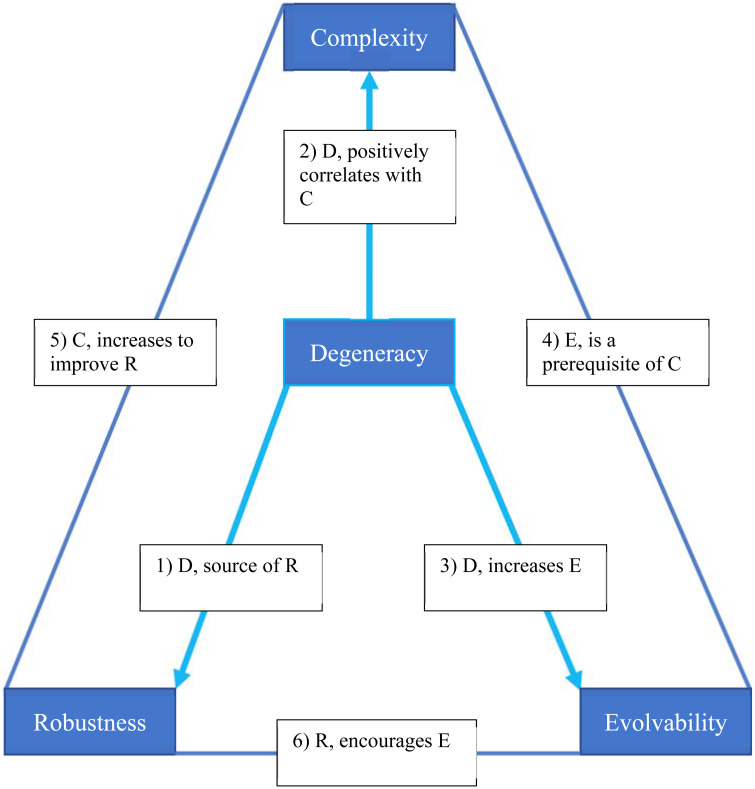
An example of the functional relationships between degeneracy, complexity, robustness and evolvability, after Whitaker and Bender 2009.

When degeneracy is low in a complex adaptive system, many different elements can affect output in a similar way, but do not have independent effects. In highly degenerate systems, many different elements can affect output in a similar way, but also have independent effects, and may be recruited by different systems, to achieve different tasks. Edelman and Gally (2001) showed that degeneracy is expressed at many different levels of biological organisation, from the genetic code itself, through levels including protein folding, metabolism, diet, cell signalling, synaptic plasticity, body motion, skill acquisition, motor learning and language. Thus, they argued, degeneracy is both necessary for, and an inevitable outcome of, natural selection. Based on Complex Adaptive Systems modelling, Whitaker and Bender (2009) argued that at all levels from the genome to locomotor behaviour, degeneracy is a source of robusticity, positively correlated with complexity, and yet increases adaptability and evolvability. In turn, evolvability is a requirement for complexity, and increased complexity enhances robustness (Figure 1). Whitaker shows that this relationship is demonstrated by cryptic genetic variation, which is made possible by system robusticity in natural selection, as well as by genetic regulatory networks and thus evolution itself. In simulation experiments, Whitaker and Bender (2009) showed that purely redundant systems have very low evolvability. In contrast, degenerate, and thus only partially redundant, systems display orders-of-magnitude-higher evolvability.

Thus, the feet of primates, by retaining a highly degenerate structure, optimise both robustness in complex environments (such as the mixed woodland/grassland environments occupied by early hominins) and evolvability in cases of climatic and environmental change, which characterised much of East and South Africa since the late Miocene. We argue that rather than interpreting the structure of early hominin feet as a mosaic of human-like and nonhuman-ape-like characteristics, degeneracy allows us to conceptualise how feet, such as those of *A. sediba*, are admirably adapted to unstable environmental conditions, while able to sustain capability within the range of immediately available habitats.

Similarly, the degeneracy equally evident in the hand would have allowed early hominins to use the hand both in a support role, in arboreal contexts, and in manipulation, in terrestrial contexts. When performing in complex, dynamic performance environments, Rein et al. (2010) showed that skilled basketball players, for example, can exploit two to six distinct patterns of joint coordination to shoot at the same target from different distances. Seifert et al. (2014) demonstrated that expert ice climbers also show higher levels of system degeneracy, namely a greater range of coordination patterns, than novices, allowing them to exploit grip opportunities in the ice much more effectively. Similar phenomena have been observed when expert musicians are compared with novices (Verrel et al., 2013; Slater 2020). Such studies have substantial relevance to the skills involved in stone tool manufacture. Bardo et al. (2018) used modelling approaches to assess biomechanical potential for tool-related behaviours, observing that the orang-utan hand performed poorly compared with that of humans and gorillas, requiring higher muscle forces for a similar range of motion. They found that the hand of *A. sediba*, however, would have had the potential for stone tool use, and perhaps manufacture, suggesting that prehensive capability had become more important to these individuals than stable grasping of larger vines and branches, consistent with the suggestion of Bardo et al. (2018). Unspecialised hands appear to have higher capacity for fine prehension, but, by analogy to the above, we suggest also for development of expertise, in individual tool-making hominins. Thus, humans who begin to knap tools would have low degeneracy in terms of joint coordination, only enhancing degeneracy progressively as their expertise improves.

High variability in morphology is an in-built mechanism of natural selection, which, in hominoids, with their unspecialised extremities, facilitated a sustained capacity to use affordances of both arboreal and terrestrial terrains in the changing environments of the late Miocene and Pliocene. Even for late Plio-Pleistocene environments, where substantial savannah had appeared, Anton et al. (2014) noted the potential role of highly diverse habitats, affecting speciation opportunities favouring hominin adaptive versatility. Local habitat mosaicism, which would also offer high biodiversity, is typical of protohominins and early hominin environments, as in the palaeoenvironments attributed to *Sahalanthropus tchadensis* (Brunet et al., 2002; Brunet, 2002), 7 Ma, from Chad, which are described as being akin to the Okavango Delta (Central Kalahari, Botswana; Brunet, 2010). Such complex environments would also be expected to exert selective pressure for ‘adaptive versatility’, or as we refer to it, locomotor plasticity, driven by inherent system degeneracy.

## 7. Conclusions

Our analysis and commentary suggest that variability and variance in the fossil record present a history in bone of the different kinematic solutions available in highly degenerate, complex and adaptive, primate postcranial systems. In this paper we have suggested how the concept of degeneracy could underpin a new interpretive paradigm for explaining observed variability in fossil elements, fortifying interpretations of possible locomotor behaviours. We also suggest that using a degeneracy framework to conceptualise human movement systems as complex adaptive systems encourages a move away from a reductionist perspective in studying neuroanatomical components in isolation of the affordances available in the surrounding environment. The current frequency of use of the term ‘mosaicism’ demonstrates the unfamiliarity that palaeoanthropologists have with the concept of *variability*, at many levels of analysis. This is understandable, considering the small sample sizes available, and the historically negative connotations of variability. Nevertheless, given the complexity of the foot, we find it essential to anchor locomotor and functional interpretations of the observed variability in systems biology, specifically harnessing the framework of *neurobiological degeneracy*. The observed variability in foot bone morphology across great apes and in early hominin feet should similarly be interpreted in terms of high levels of degeneracy in the foot complex, selecting for locomotor plasticity.

We have provided evidence from the foot – one of only two structures in the body that can interact directly in contact with the environment during locomotory movement – that variability is inherent to functional biological movement, enabling adaptable and flexible locomotor behaviours in rich and varied terrains.

We have argued that the observed variability in the fossil record can be understood more completely when using the theory of neurobiological degeneracy. Degeneracy can explain not only the observed variability in biomechanical parameters, but also the observed morphological overlap in hominin fossil and primate species. Functional variability in the foot has been a selective target in hominoids as a whole, and in hominins in particular, as a key component of overall locomotor plasticity and degeneracy.

The main implications for the palaeontological literature are that using the theory of degeneracy provides a cohesive, interpretive framework that may be beneficial in: (a) (re)interpreting the observed variability and overlap between hominoid fossil and extant species, and (b) providing a more robust understanding of evolutionary pressures that shaped structurally complex organs, such as the hominoid and hominin foot, and the mechanisms which retain both evolvability and robusticity, than those currently conceptualised as mosaicism.

## Data Availability

This is a review paper and does not rely on primary data
